# What is the impact of knee morphology on posterior cruciate ligament avulsion fracture in men and women: a case control study

**DOI:** 10.1186/s12891-021-03984-5

**Published:** 2021-01-21

**Authors:** Ning Fan, Yong-chen Zheng, Lei Zang, Cheng-gang Yang, Shuo Yuan, Peng Du, Yan-mei Liu, Qing Zhao, Jin-wei Wang

**Affiliations:** 1 Department of Orthopedics, Beijing Chaoyang Hospital, Capital Medical University, Beijing, China; 2 Department of Orthopedics, Beijing Shunyi District Hospital, Beijing, China

**Keywords:** Posterior cruciate ligament, Avulsion fracture, MRI, Risk factor, Morphology

## Abstract

**Background:**

Several studies on the relationship between morphological parameters and traumatic diseases of the knee have already been conducted. However, few studies focused on the association between knee morphology and posterior cruciate ligament (PCL) avulsion fracture in adults. The objective of this study was to evaluate the impact of knee morphology on PCL avulsion fracture.

**Methods:**

76 patients (comprised 40 men and 36 women) with PCL avulsion fracture and 76 age- and sex-matched controls without PCL avulsion fracture were studied from 2012 to 2020. MRI measurements of the knee were acquired in the sagittal, coronal, and axial planes. The assessed measurements including intercondylar notch width index, coronal tibial slope, and medial/lateral posterior tibial slopes were compared between men and women, and between case and control groups respectively using independent sample t-tests. In addition, binary logistic regression analyses were used to identify independent risk factors of PCL avulsion fracture.

**Results:**

Except notch width index (coronal) (*p* = 0.003) in the case groups, there was no statistical difference in the assessed measurements including notch width index (axial), coronal tibial slope, medial posterior tibial slope, and lateral posterior tibial slope between men and women in the case and control groups (*p* > 0.05). When female patients were analyzed, the notch width index (coronal) was significantly smaller (*p* = 0.0004), the medial posterior tibial slope (*p* = 0.018) and the lateral posterior tibial slope (*p* = 0.033) were significantly higher in the case group. The binary logistic regression analysis showed that the notch width index (coronal) (B = -0.347, OR = 0.707, *p* = 0.003) was found to be an independent factor of PCL avulsion fracture. However, none of the assessed measurements was found to have a statistical difference between the case and control groups in men (*p* > 0.05).

**Conclusions:**

Notch width index (coronal), medial posterior tibial slope, and lateral posterior tibial slope were found to affect PCL avulsion fracture in women, but no such measurements affected the PCL avulsion fracture in men. Furthermore, a smaller notch width index (coronal) in women was found to be a risk factor in PCL avulsion fracture.

## Background

The posterior cruciate ligament (PCL) is the strongest ligament in the knee, and injuries to the PCL occur less commonly than in the anterior cruciate ligament (ACL). PCL avulsion fracture(s) represent a specific form of PCL injury and are less common than the typical intrasubstance PCL tear [1]. It often results from high-energy mechanisms in adults, such as a direct blow to the tibia with the knee in flexion (motor vehicle collision) or severe hyperextension (sports-related trauma) [2]. However, as more people engage in sports, these injuries are likely to increase in number. MRI is an efficient and noninvasive modality that can reliably diagnose PCL avulsion fracture and evaluate knee morphology. It is generally agreed that a displaced PCL avulsion fracture should be anatomically reduced and fixed. Both open and arthroscopic methods of fixation have been reported to produce good outcomes [3–5].

Nowadays, several studies on the relationship between morphological parameters and traumatic diseases of the knee have already been conducted [6, 7]. Many studies have found different relationships between measurements of knee geometry and ACL injury, such as stenotic intercondylar notch type [8], intercondylar notch volume [9], medial tibial slope [10], lateral tibial slope [11], and tibial slopes (both bony and meniscal) [12]. Similarly, Song et al. reported that increased medial meniscal slope was associated with a greater risk of ramp lesion of the meniscus in noncontact ACL injury [13]. Kocher et al. performed a comparison between tibial spine avulsion fractures and midsubstance ACL disruptions in skeletally immature patients and found a correlation between decreased notch width index (NWI) and midsubstance ACL disruption [14]. Recently, van Kuijk et al. reported that a smaller and more sharply angled intercondylar notch and a more flattened tibial eminence are related to PCL rupture [15]. In addition, Bernhardson et al. reported that decreased tibial slope appears to be a risk factor for primary PCL injury [16]. Furthermore, we are still unclear whether there is an association between magnetic resonance imaging (MRI) measurements of the knee and PCL avulsion fracture as a specific form of PCL injury in adults.

A higher prevalence of ACL injury in females has been recognized by numerous studies over the last decade. In addition, the differences in anatomic risk factors of ACL injury between men and women have been widely studied [17–20]. Similarly, we assumed that the risk factors of PCL avulsion fracture may differ between men and women, and the risk factors in men and women should be therefore analyzed respectively rather than mixing or comparing the genders.

Therefore, we designed this case-control study to evaluate the impact of knee morphology on PCL avulsion fracture and determine risk factors in men and women respectively. Those measurements include a notch width index, medial/lateral posterior tibial slopes, and coronal tibial slope. The hypotheses of this study were that certain parameters were highly associated with PCL avulsion fracture and that the risk factors differed between men and women.

## Methods

### Study Design

In the present study, we included 76 patients who suffer a direct blow to the tibia with the knee in flexion or severe hyperextension and were diagnosed with PCL avulsion fracture by MRI performed at our institution from 2012 to 2020. The study group comprised 40 men and 36 women whose mean age was 53.95 years (range, 27–82 years). There were 33 patients with the complication of posterior root tears of the medial meniscus. The exclusion criteria for this study were age < 18 years, previous knee surgery, MRI obtained at an outside hospital or with insufficient quality (evident blurring because of patient movement during MRI), tibia plateau fracture, old PCL avulsion fracture (more than 3 months), ACL avulsion fracture, and ACL injury. Age- and sex-matched controls (comprised 40 men and 36 women) were selected in a 1:1 ratio for comparison. Moreover, controls underwent MRI for knee injuries() such as (a) direct blow to the tibia with the knee in flexion and severe hyperextension. The investigation was approved by the hospital’s institutional review board, and subjects provided informed consent prior to participation.

Tibial and femoral morphological characteristics were measured on MRI studies using Philips Achieva 1.5-T and 3-T MRI system (Philips Medical Systems) in both groups. MRI measurements of the knee were acquired in the sagittal, coronal, and axial planes. The sagittal plane included the T1-weighted and T2-weighted phases. All images were reviewed by 2 senior orthopedic surgery residents. All measurement collections were performed by two trained orthopedic surgeons using DICOM (version 3.1) viewer software (Neusoft PACS/RIS) [13].

Morphological measurements of the coronal sequence include intercondylar notch width, medial condylar width, lateral condylar width, condylar width, coronal tibial slope, and notch width index. Morphological measurements of the axial sequence include intercondylar notch width, medial condylar width, lateral condylar width, condylar width, and notch width index. Morphological measurements of the sagittal sequence include medial posterior tibial slope and lateral posterior tibial slope. The assessed measurements including intercondylar notch width index, medial/lateral posterior tibial slopes, and coronal tibial slope were compared between men and women in the case and control groups, and between case and control groups in men and women respectively.

### Morphological measurements

**Fig. 1:**
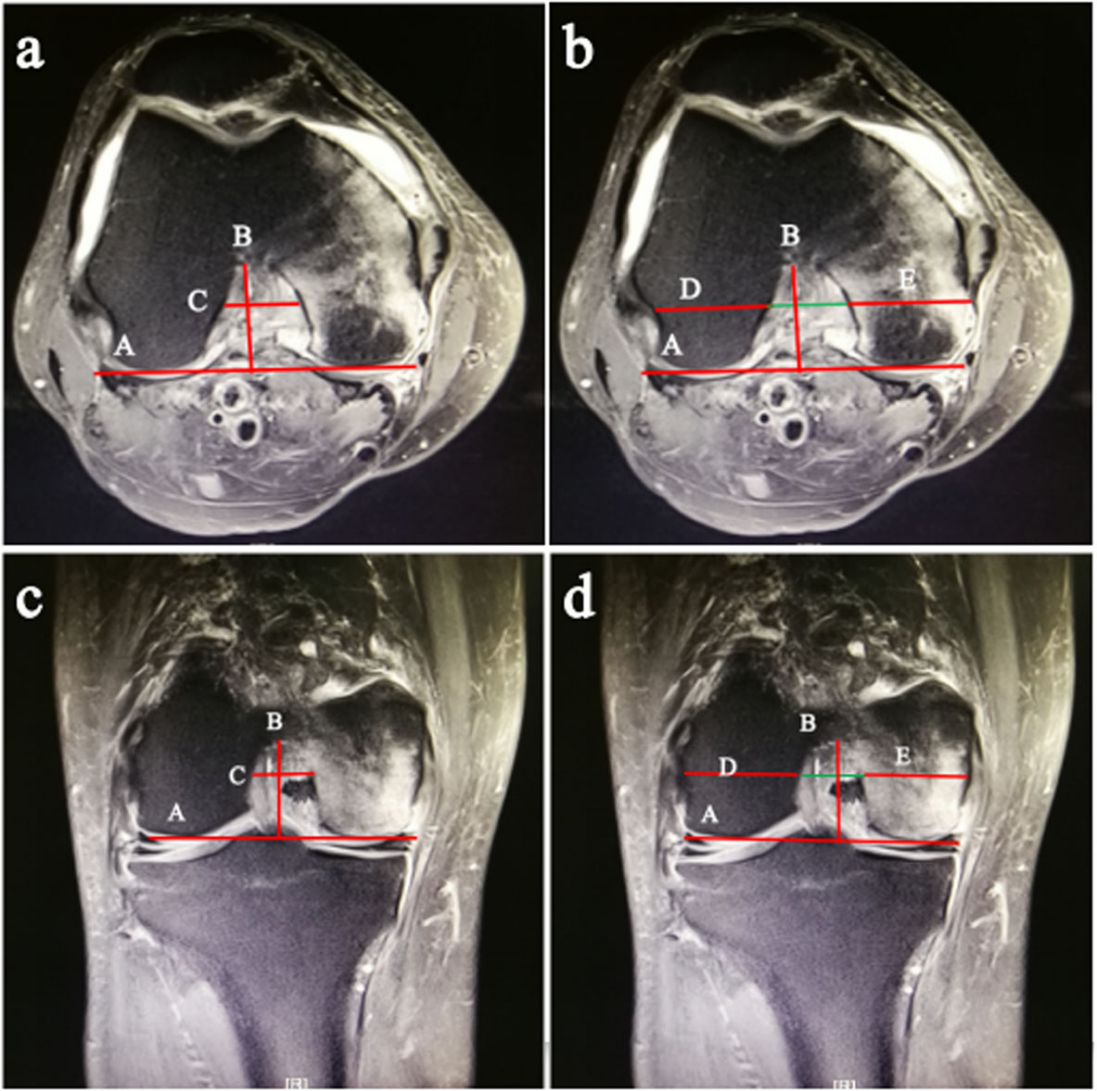
Magnetic resonance images depicting the method used to obtain the femoral measurements in the axial plane. **a** The intercondylar height (line B) was the distance from the top of the intercondylar notch to the bicondylar line (line A). The intercondylar width (line C) was obtained at the anterior third of the intercondylar height (line B). **b** At the same level, the width of the lateral and medial condyles (line D, E) was obtained. The same method was used to collect these measurements in the coronal plane (**c** and **d**)

Femoral measurements were obtained for the coronal and axial planes, as described by Stein et al. and Alentorn et al. [21, 22]. In both planes, a cut showing the popliteal groove was used to measure the femoral morphological parameters. For the axial plane, a posterior bicondylar line and its perpendicular line from the top of the intercondylar notch were determined. The intercondylar height (line B) was the distance from the top of the intercondylar notch to the bicondylar line (line A) (Fig. 1a). The intercondylar width (line C) was obtained at the anterior third of the intercondylar height (line B) in the axial plane (Fig. 1a). At the same level, the width of the lateral and medial condyles (line D, E) was obtained (Fig. 1b). The condylar width was considered the sum of the intercondylar, medial condylar, and lateral condylar widths. The same method was used to collect these measurements in the coronal plane (Fig. 1c and d). Moreover, the notch width index was the ratio of intercondylar notch width and condylar width.

**Fig. 2:**
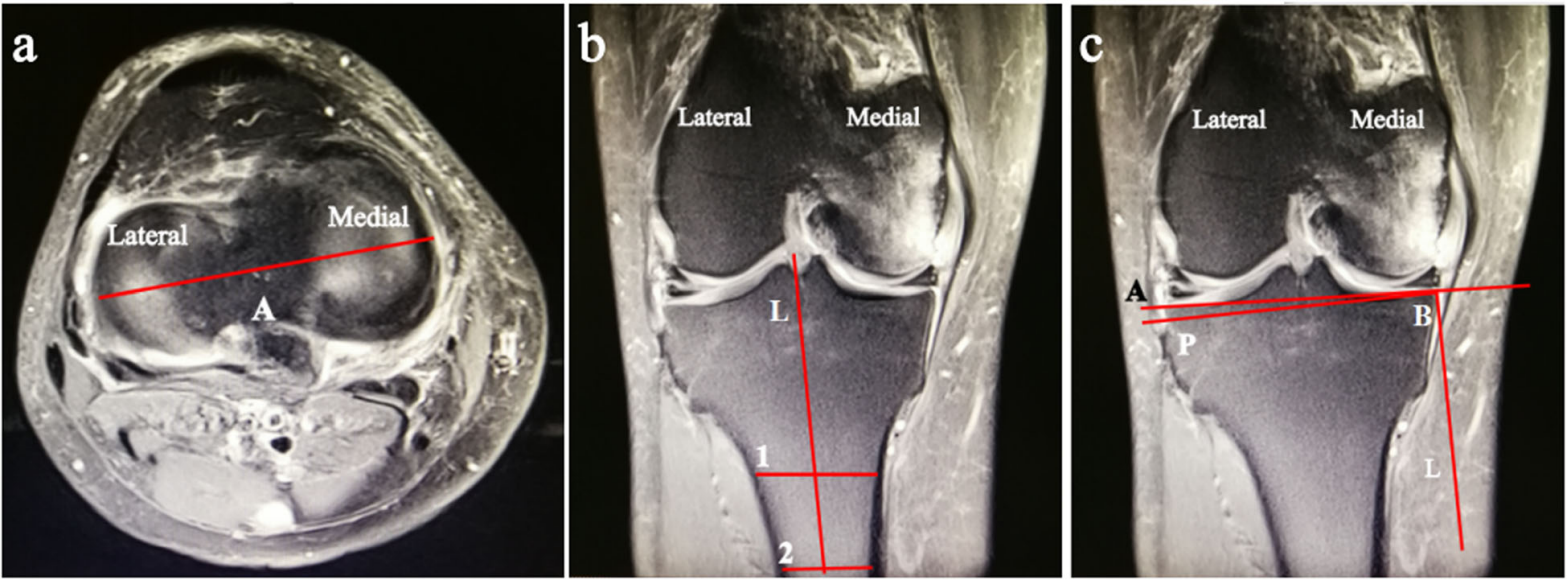
Magnetic resonance images depicting the method used to obtain the coronal slopes. **a** This shows the transverse plane passing through the tibiofemoral joint and showing the dorsal aspect of the tibial plateau. Line A shows the location of the coronal plane that passed closest to the centroid of the tibial plateau. **b** The longitudinal axis (line L) of the tibia was obtained by determining the midpoint of the medial-to-lateral width of the tibia at two points located as distally in the image as possible (locations 1 and 2). **c** The coronal tibial slope was the angle between a line joining the peak points on the medial and lateral aspects of the plateau (points A and B) and line P perpendicular to line L

The coronal slopes of the tibial plateaus were obtained as described by Hashemi et al. [23]. The first step was to identify a transverse plane passing through the tibiofemoral joint and showing the dorsal aspect of the tibial plateau (Fig. 2a). In this transverse image, the coronal plane that passed closest to the centroid of the tibial plateau was identified. The location of this plane is represented by the horizontal line in Fig. 2a, and the corresponding coronal plane is shown in Fig. 2b. Next, the orientation of the longitudinal axis of the tibia was determined. This was obtained by determining the midpoint of the medial-to-lateral width of the tibia at two points located approximately 4 to 5 cm apart and as distally in the image as possible (locations 1 and 2 in Fig. 2b). The extended line connecting the two midpoints represents the longitudinal axis of the tibia in the coronal plane. The coronal tibial slope was then measured as the angle between a line joining the peak points on the medial and lateral aspects of the plateau (points A and B in Fig. 2c) and the line perpendicular to the longitudinal axis (line P in Fig. 2c). If point A is below the perpendicular line P, then the slope was designated as negative.

**Fig. 3:**
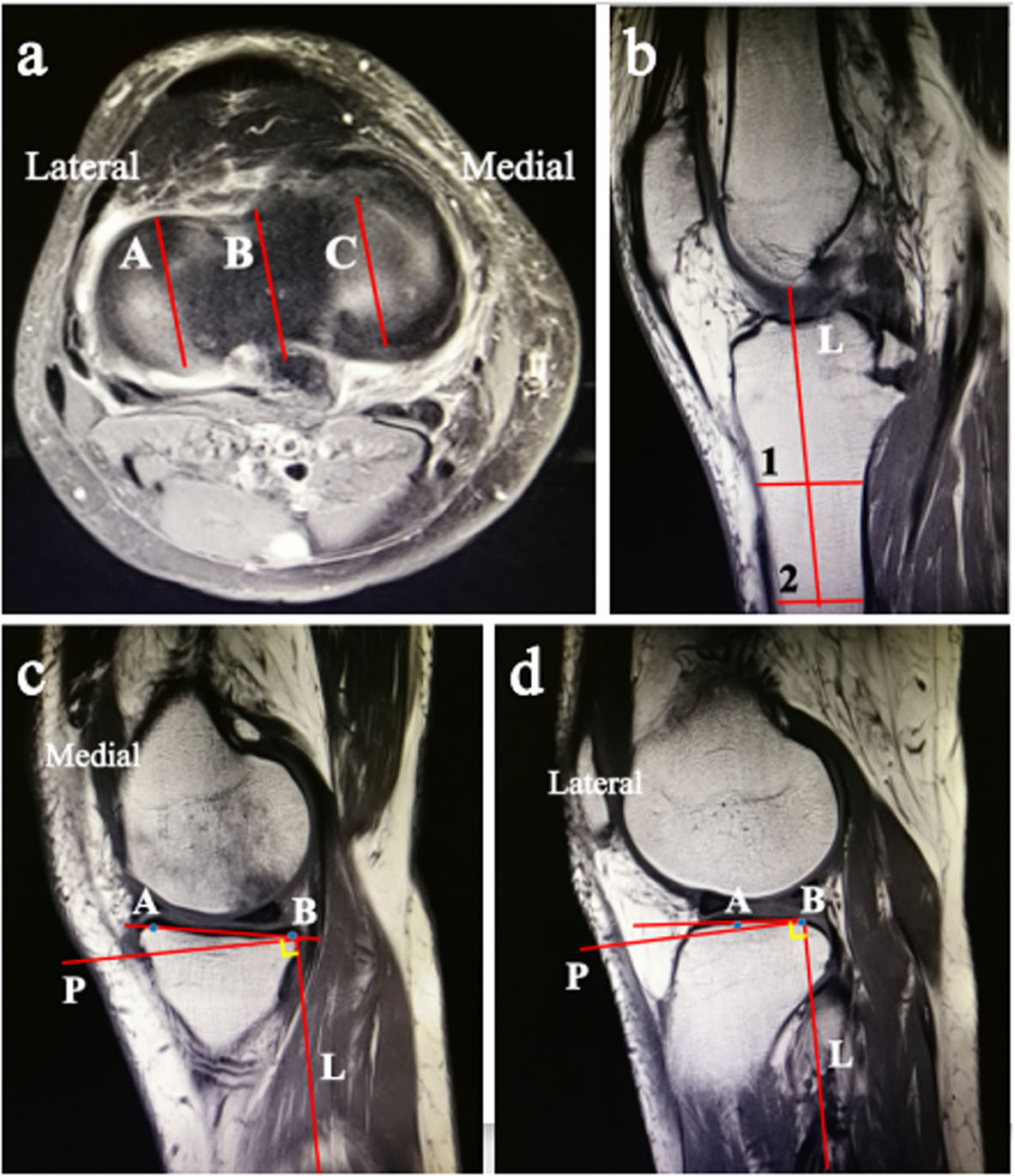
Magnetic resonance images depicting the method used to obtain the sagittal slopes of the medial and lateral tibial plateaus. **a** This shows the transverse plane passing through the tibiofemoral joint and showing the dorsal aspect of the tibial plateau. Lines A and C show the corresponding locations of the sagittal planes in the medial and lateral tibial plateaus. Line B in the same figure shows the approximate location of the sagittal plane that was used to determine the orientation of the longitudinal axis. **b** The longitudinal axis (line L) of the tibia was obtained by determining the midpoint of the anterior and posterior widths of the tibia at two points located as distally in the image as possible (locations 1 and 2). **c** The medial tibial slope was the angle between a line joining the peak points on the medial plateau (points A and B) and line P perpendicular to line L. **d** The lateral tibial slope was the angle between a line joining the peak points on the lateral plateau (points A and B) and line P perpendicular to line L

The sagittal slopes of the medial and lateral tibial plateaus were also obtained as described by Hashemi et al. [23]. In Fig. 3a, lines A and C show the corresponding locations of the sagittal planes in the medial and lateral tibial plateau. Line B shows the approximate location of the sagittal plane that was used to determine the orientation of the longitudinal axis. We used the plane that clearly showed the orientation of the tibia (Fig. 3b). As before, the anterior and posterior cortices of the tibial shaft at two points located approximately 4 to 5 cm apart and distal in the image (locations 1 and 2 in Fig. 3b) were determined. The midpoints of the lines representing the anterior-posterior thickness of the tibia were found, and the longitudinal axis (L) was constructed. The longitudinal axis was then reproduced in the medial plane as shown in Fig. 3c. The peak anterior and posterior points on the tibial plateau were identified (points A and B). The slope of the line extending through these two points represented the medial tibial slope and was measured with respect to the axis (P) perpendicular to the longitudinal axis (L). A similar approach was used to determine the lateral tibial slope (Fig. 3d). Because the peak anterior points on the tibial plateau are proximal to the peak posterior points in both Figs. 3c and d, the slopes of the medial and lateral tibial plateaus would be positive according to the existing convention.

To calculated intraclass correlation coefficients (ICC) which demonstrating the inter- and intraobserver reproducibility, 20 patients (10 from the case group and 10 from the control group) were randomly included to be analyzed [11, 13]. All the morphological measurements were conducted by two trained orthopedic surgeons blinded to the project independently, and one orthopedic surgeon seperately for twice by two weeks apart at least. ICC values ≥ 0.8 were defined as good, ≥ 0.9 were definded as excellent.

### Statistical analysis

All analyses were performed by using SPSS version 22.0. Independent sample t-tests were performed to compare the measurements between men and women in the case and control groups. The same analysis was conducted between the case and control groups. Separate analyses were completed for men and women. Additionally, binary logistic regression analysis was performed to determine the independent predictors of PCL avulsion fracture. Separate analyses were completed for men and women. The inter- and intraobserver reproducibilities were determined by the ICCs. Statistical significance was set at p < 0.05.

## Results

The intra- and interobserver reliabilities for the knee morphological features are presented in Table 1. The intraobserver ICC for all variants ranged from 0.699 to 0.949. Similarly, the interobserver ICC was 0.778 to 0.929, which revealed a strong agreement between observers for all measurements.

**Table 1:** Intra- and inter-observer intraclass correlation coefficients (ICCs) for all variants of 20 random patients

Variable	Intra-observer ICC	Inter-observer ICC
Intercondylar notch width (axial)	0.934	0.894
Medial condylar width (axial)	0.949	0.913
Lateral condylar width (axial)	0.874	0.873
Intercondylar notch width (coronal)	0.802	0.860
Medial condylar width (coronal)	0.886	0.929
Lateral condylar width (coronal)	0.948	0.899
Coronal tibial slope	0.882	0.778
Medial posterior tibial slope	0.699	0.867
Lateral posterior tibial slope	0.705	0.914

The radiographic measurements of the femoral condyle in the axial and coronal sequences were shown in Table 2. We found that intercondylar notch widths (axial and coronal), medial condylar widths (axial and coronal), lateral condylar widths (axial and coronal), and condylar widths (axial and coronal) of men were significantly higher than those of women in the case and control groups (*p* < 0.05). However, except notch width index (coronal) (*p* = 0.003) in the case groups, there was no statistical difference in the assessed measurements including notch width index (axial), coronal tibial slope, medial posterior tibial slope, and lateral posterior tibial slope between men and women in the case and control groups (*p* > 0.05) (Table 3).

**Table 2:** The radiographic parameters of the femoral condyle in the axial and coronal sequences

Variable	Case group			Control group		
	Women (*n* = 36)	Men (*n* = 40)	*P* Value	Women (*n* = 36)	Men (*n* = 40)	*P* Value
Intercondylar notch width (axial), mm	18.25 ± 1.791	20.89 ± 2.027	0.000^*^	17.76 ± 1.538	19.62 ± 2.156	0.000^*^
Medial condylar width (axial), mm	24.59 ± 1.768	27.20 ± 1.830	0.000^*^	23.77 ± 1.950	27.48 ± 2.285	0.000^*^
Lateral condylar width (axial), mm	25.39 ± 2.963	30.65 ± 2.709	0.000^*^	25.49 ± 1.612	29.95 ± 2.759	0.000^*^
Condylar width (axial), mm	68.24 ± 4.037	78.74 ± 4.195	0.000^*^	67.02 ± 2.567	77.05 ± 4.573	0.000^*^
Intercondylar notch width (coronal), mm	16.33 ± 1.667	20.05 ± 2.709	0.000^*^	17.65 ± 1.892	19.09 ± 1.849	0.001^*^
Medial condylar width (coronal), mm	25.39 ± 2.593	28.03 ± 1.826	0.001^*^	24.81 ± 1.370	28.32 ± 2.615	0.000^*^
Lateral condylar width (coronal), mm	29.18 ± 2.910	32.92 ± 2.351	0.000^*^	27.81 ± 2.720	31.49 ± 3.067	0.000^*^
Condylar width (coronal), mm	70.90 ± 4.249	81.00 ± 4.080	0.000^*^	70.20 ± 3.530	78.90 ± 4.880	0.000^*^

Asterisks indicate statistical significance (*p* < 0.05)

**Table 3:** The five radiographic parameters of the case and control groups

Variable	Case group			Control group		
	Women (*n* = 36)	Men (*n* = 40)	*P* Value	Women (*n* = 36)	Men (*n* = 40)	*P* Value
Notch width index (axial)	0.27 ± 0.026	0.27 ± 0.026	0.945	0.27 ± 0.022	0.26 ± 0.028	0.078
Notch width index (coronal)	0.23 ± 0.022	0.25 ± 0.028	0.003^*^	0.25 ± 0.028	0.24 ± 0.025	0.160
Coronal tibial slope, °	3.75 ± 3.333	3.20 ± 2.356	0.414	4.06 ± 2.651	3.58 ± 2.561	0.425
Medial posterior tibial slope, °	8.17 ± 4.032	8.55 ± 2.952	0.635	6.25 ± 2.489	7.13 ± 3.784	0.234
Lateral posterior tibial slope, °	7.97 ± 4.754	8.50 ± 4.182	0.608	5.78 ± 3.735	7.35 ± 4.300	0.095

Asterisks indicate statistical significance (*p* < 0.05)

In women, we found that the notch width index (coronal) was significantly smaller, the medial posterior tibial slope and the lateral posterior tibial slope were significantly higher in the case group (0.23 ± 0.022 compared with 0.25 ± 0.028, *p* = 0.0004; 8.17° ± 4.032° compared with 6.25° ± 2.489°, *p* = 0.018; 7.97° ± 4.754° compared with 5.78° ± 3.735°, *p* = 0.033; respectively) (Table 4). In contrast, when male patients were analyzed, no assessed measurement was found to have a statistical difference between the case and control groups (*p* > 0.05) (Table 4).

**Table 4:** The radiographic parameters in women and men

Variable	Women			Men		
	Cases (*n* = 36)	Controls (*n* = 36)	*P* Value	Cases (*n* = 40)	Controls (*n* = 40)	*P* Value
Notch width index (axial)	0.26 ± 0.026	0.26 ± 0.022	0.692	0.27 ± 0.026	0.26 ± 0.028	0.069
Notch width index (coronal)	0.23 ± 0.022	0.25 ± 0.028	0.0004^*^	0.25 ± 0.028	0.24 ± 0.025	0.477
Coronal tibial slope, °	3.75 ± 3.333	4.06 ± 2.651	0.668	3.20 ± 2.356	3.58 ± 2.561	0.497
Medial posterior tibial slope, °	8.17 ± 4.032	6.25 ± 2.489	0.018^*^	8.55 ± 2.952	7.13 ± 3.784	0.064
Lateral posterior tibial slope, °	7.97 ± 4.754	5.78 ± 3.735	0.033^*^	8.50 ± 4.182	7.35 ± 4.300	0.229

Asterisks indicate statistical significance (*p* < 0.05)

In women, all predictors were included in a binary logistic regression analysis to determine the independent factors of PCL avulsion fracture (Table 5). Notch width index (coronal) (B = -0.347, OR = 0.707, *p* = 0.003) was found to be an independent factor of PCL avulsion fracture.

**Table 5:** Independent predictive factors of PCL avulsion fracture in women

Variable	B	SE	Wald	*p* Value	Odds Ratio	95 % CI for EXP (B)	
						Lower limit	Upper limit
Notch width index (coronal)	-0.347	0.117	8.776	0.003^*^	0.707	0.562	0.889
Medial posterior tibial slope	0.106	0.107	0.989	0.320	1.112	0.902	1.372
Lateral posterior tibial slope	0.054	0.084	0.413	0.521	1.056	0.895	1.245

Asterisks indicate statistical significance (*p* < 0.05)

## Discussion

The main finding of the present study is that there are significant differences in bony morphology between female patients with PCL avulsion fracture and those without. Our research showed that the notch width index (coronal) was significantly smaller, while the medial posterior tibial slope and lateral posterior tibial slope were significantly higher in the female patients with PCL avulsion fracture. What’s more, a smaller notch width index (coronal) in women was found to be related to PCL avulsion fracture. However, no such measurements affected the PCL avulsion fracture in men. These results also demonstrated that the knee morphological measurements, which were mostly associated with the risk of sustaining a PCL avulsion fracture, differ between men and women.

Many studies have been performed previously to identify potential risk factors in ACL and PCL injuries, and the femoral notch has been the main topic of interest. Domzalski et al. [24] have suggested that a smaller notch width index can be correlated to an ACL rupture. Recently, van Kuijk et al. reported that a smaller and more sharply angled intercondylar notch is related to PCL rupture [15]. Interestingly, the findings of that study were consistent with the findings in our present study. In our study, notch width index (coronal) was also found to be an independent factor in women, This means that a smaller notch width index (coronal) may render the knee more susceptible to a PCL avulsion fracture. Previous research has shown that patients with smaller intercondylar notch also have smaller ACL and PCL [25, 26]. Moreover, a smaller PCL can resist less force than a larger PCL [15]. Therefore, a patient with a smaller intercondylar notch also has a smaller PCL and tibial footprints of PCL, while the smaller tibial footprints can only bear smaller external stress, which may increase the risk of the avulsion fracture. In addition, a notch width index (coronal) as it relates to the femoral footprint of PCL could influence the direction of PCL. A smaller notch width index (coronal) may lead to the direction of PCL close to the center of the knee and it may increase the stress of the tibial footprints of PCL, resulting in an increased risk of injury with a direct blow to the tibia with the knee in flexion. In this regard, further research is needed to better understand this relationship.

Previous studies have shown that the posterior tibial slope is significantly correlated with non-contact ACL and PCL injuries. A larger posterior tibial slope increases the risk of ACL rupture [10–12], while a smaller posterior tibial slope increases the risk of PCL rupture [16]. The mechanism of injury in ACL rupture shows an increase of tibial slope, leading to an increase of tibial forward displacement and stress in the ACL with weight-bearing activity [13, 27]. Similarly, under conditions where the tibia bears the axial load and backward external force, the reduction of the tibial slope leads to an increase of the stress in the PCL which leads to rupture. Therefore, the increase of the tibial slope has a protective effect on the PCL, and the researchers found that a 1-degree increase in the posterior tibial slope decreased PCL force by 6 N [28–31]. Bernhardson et al. confirmed the above opinions and they found that a decreased posterior tibial slope was associated with patients who have PCL tears [16].

In women, our results showed that the medial posterior tibial slope and the lateral posterior tibial slope were significantly higher in the case group women, which seems to contradict the study of Bernhardson. However, those two studies have different research subjects. PCL ruptures occur in the ligament portion, while PCL avulsion fractures occur in the bone tissue at the tibial insertion of the PCL. We believe that the posterior tibial slope plays an important role in the occurrence of these two conditions. When a sufficiently large retrograde force is applied to the proximal tibia, PCL rupture is prone to occur when the tibial slope is smaller. However, a PCL avulsion fracture is more prone to occur when the tibial slope is larger. Other factors such as the acceleration of direct force and the degree of osteoporosis may also play important roles in the occurrence of PCL avulsion fractures. Therefore, further studies are needed to verify the above hypothesis, such as a comparative study on the morphological parameters of the knee joint, especially the posterior tibial slope, between the PCL rupture group and the PCL avulsion fracture group.

Despite a considerable scientific effort to optimize surgical treatment, PCL injuries still cause a high degree of health impairment and involve high economic costs. This has served as the motivation for studies that focused on determining potential risk factors associated with PCL avulsion fracture. Because of the low prevalence of PCL avulsion fracture, screening only to determine the risk of sustaining a PCL avulsion fracture would likely not be cost efficient. However, several studies on the relationship between morphological measurements and traumatic diseases of the knee including ACL injury [8–12], ramp lesion of meniscus [13], and tibial spine avulsion fractures [14], PCL injury [15, 16], have already been conducted, and more risk factors have been associated with knee-related sports injuries. All of these risk factors could be combined as a menu to find and help patients at risk for different types of knee injury [15]. For example, patients with smaller notch width index are more prone to ACL and PCL injuries, and PCL avulsion fractures. We think that more similar studies should be carried out in the future to discover more risk factors of other knee injuries so as to expand and enrich this menu. Furthermore, understanding knee morphology may have implications for PCL avulsion fracture mechanism, strategies for injury prevention, and perhaps treatment and prognosis.

Previous studies mainly focused on the surgical technique or clinical outcomes of this injury [3–5]. To our knowledge, our study is the first to identify the association between MRI measurements of the knee and PCL avulsion fracture and find the independent factor that may help explain the risk of PCL avulsion fracture and the predisposition of patients toward knee injury. The current report suggested that there exist independent risk factors of smaller notch width index (coronal) in women, which may render the knee more susceptible to PCL avulsion fracture. In addition, we believed that MRI was superior to computed tomography (CT) and radiographs in measuring morphological parameters. The advantages of MRI include the ability to visualize the surface geometry of the articular cartilage, which allows better visibility of the functional point of the tibial slope, absence of ionizing radiation, and excellent soft tissue contrast. Furthermore, MRI has been proven as the gold standard method of choice for noninvasive evaluation of ligament injuries, including those of the ACL and PCL, which can be used to exclude patients with complicated ACL or PCL injuries.

Our study had several limitations. First, this was a retrospective study, and the small sample size might have led to a selection bias. Second, while patients were matched for age and sex, they were not matched for height, weight, and levels and styles of external force. The absence of this information may confound our data. Third, some measurements could not be performed because of the fracture. For instance, the angle consisting of the superior and posterior margins of the tibial intercondylar eminence in the sagittal sequence, which we called the posterior angle of intercondylar eminence, may be strongly associated with PCL avulsion fracture. Fourth, age may be an important factor. The femoral intercondylar notch may have bony hyperplasia, and the measurements of the intercondylar notch showed a difference as age increased. However, we did not take age into account in our study. Lastly, 33 patients had a complication of posterior root tears of the medial meniscus in the case group. However, the menisci play a role in the anteroposterior stability of the knee joint and may influence the functional tibial slope [12, 32]. In the present study, we ignored the effects of the menisci on the tibial slope.

## Conclusions

Notch width index (coronal), medial posterior tibial slope and lateral posterior tibial slope were found to affect PCL avulsion fracture in women, but no such measurements affected the PCL avulsion fracture in men. Furthermore, a smaller notch width index (coronal) in women was found to be a risk factor of PCL avulsion fracture.

## Data Availability

The datasets used and/or analysed during the current study are available from the corresponding author on reasonable request.

## Abbreviations

PCL: Posterior cruciate ligament
ACL: Anterior cruciate ligament
NWI: Notch width index
MRI: Magnetic resonance imaging
ICC: Intraclass correlation coefficients
CT: Computed tomography
